# Current Perspectives on Plague Vector Control in Madagascar: Susceptibility Status of *Xenopsylla cheopis* to 12 Insecticides

**DOI:** 10.1371/journal.pntd.0004414

**Published:** 2016-02-04

**Authors:** Adélaïde Miarinjara, Sébastien Boyer

**Affiliations:** 1 Unite d’Entomologie Médicale, Institut Pasteur de Madagascar, Antananarivo, Madagascar; 2 Ecole Doctorale Sciences de la Vie et de l’Environnement, Université d’Antananarivo, Antananarivo, Madagascar; University of California San Diego School of Medicine, UNITED STATES

## Abstract

Plague is a rodent disease transmissible to humans by infected flea bites, and Madagascar is one of the countries with the highest plague incidence in the world. This study reports the susceptibility of the main plague vector *Xenopsylla cheopis* to 12 different insecticides belonging to 4 insecticide families (carbamates, organophosphates, pyrethroids and organochlorines). Eight populations from different geographical regions of Madagascar previously resistant to deltamethrin were tested with a World Health Organization standard bioassay. Insecticide susceptibility varied amongst populations, but all of them were resistant to six insecticides belonging to pyrethroid and carbamate insecticides (alphacypermethrin, lambdacyhalothrin, etofenprox, deltamethrin, bendiocarb and propoxur). Only one insecticide (dieldrin) was an efficient pulicide for all flea populations. Cross resistances were suspected. This study proposes at least three alternative insecticides (malathion, fenitrothion and cyfluthrin) to replace deltamethrin during plague epidemic responses, but the most efficient insecticide may be different for each population studied. We highlight the importance of continuous insecticide susceptibility surveillance in the areas of high plague risk in Madagascar.

## Author Summary

In spite of more than 50 years of efforts to control plague, Madagascar is one of the countries with the highest plague incidence. Bubonic plague, the most encountered form, is transmitted by flea bites. The plague control and prevention policy is based on flea control with chemical insecticides. Hence the occurrence of flea resistance is of major concern in the public health context. Our research team conducted laboratory work to assess the resistance level of *Xenopsylla cheopis*, the main plague vector, to 12 insecticides. The results of this study will contribute to more focused flea population control, and therefore more efficient control of plague outbreaks.

## Introduction

Arthropod-borne diseases are a major concern worldwide. Every year more than 1 billion cases and over 1 million deaths from vector-borne diseases are estimated [1]. Most of these vectors are bloodsucking arthropods (e.g., mosquitoes, flies, fleas, ticks, lice) living in direct contact with humans or harbored by livestock or commensal animals [2–5]Arthropod-borne diseases such as malaria, chikungunya, dengue fever, Lyme disease, West Nile fever, Rift Valley fever, and plague erupt and cause substantial mortality in humans and livestock [1,5–8]. The risk posed by these diseases can be significantly reduced by the use of insecticides during a public health emergency; insecticide-based intervention can prevent an outbreak or can limit the expansion of the disease [3,5].

In the 1940s, the use of synthetic insecticides led to great improvement in the battle against disease vectors [3,9]. Consequently, the intensive use of insecticides caused selection pressure on insect populations, which developed mechanisms to survive insecticide treatments. All classes of insecticides are currently involved, and the list of pests associated with agriculture and health has been continually increasing [10]. The lack of new insecticidal compounds and the misuse or overuse of insecticides have been identified as reasons of the emergence of insecticide resistance in pests [11]. Hence, an efficient vector control policy must take in account the possibility of insecticide resistance, which can lead to a failure of the control strategy [12]. The early detection and monitoring of insecticide resistance in a vector population may positively impact intervention strategies. Until now, the main defense against resistance is close surveillance of the susceptibility of vector populations [4].

Plague, a rodent disease transmitted to human by infected flea bites, remains an important health problem in Madagascar [13]. The flea, *Xenopsylla cheopis* is the main plague vector, parasitizing black rats *Rattus rattus* that live in urban and rural housing [14]. According to the World Health Organization (WHO), the most rapid and effective method for controlling fleas is to apply an appropriate insecticide formulated as a dust or low-volume spray [15,16]. Insecticide dusting in households is the strategy adopted by the National Plague Control Program in Madagascar to control vectors and to limit the expansion of plague epidemics [17,18]. In 1947 the use of DDT (dichlorodiphenyltrichloroethane) insecticide to control fleas in Madagascar gave new hope for combatting plague. Since 1965, resistance of *X*. *cheopis* to DDT has been developing in Madagascar [19]. Later, *X*. *cheopis* populations were reported to be resistant to different families of insecticide from the early 1980s to 2000 [20–26]. More recently, amongst 32 populations of *X*. *cheopis*, only two populations were susceptible to deltamethrin, which is currently the preferred insecticide in Madagascar for flea control [27]. Hence, it is crucial to find insecticide alternatives to deltamethrin. Here we report the results of 12 different insecticide bioassays performed on 8 populations of *X*. *cheopis* previously found to be resistant to deltamethrin.

## Materials and Methods

### Flea populations

**Fig 1:**
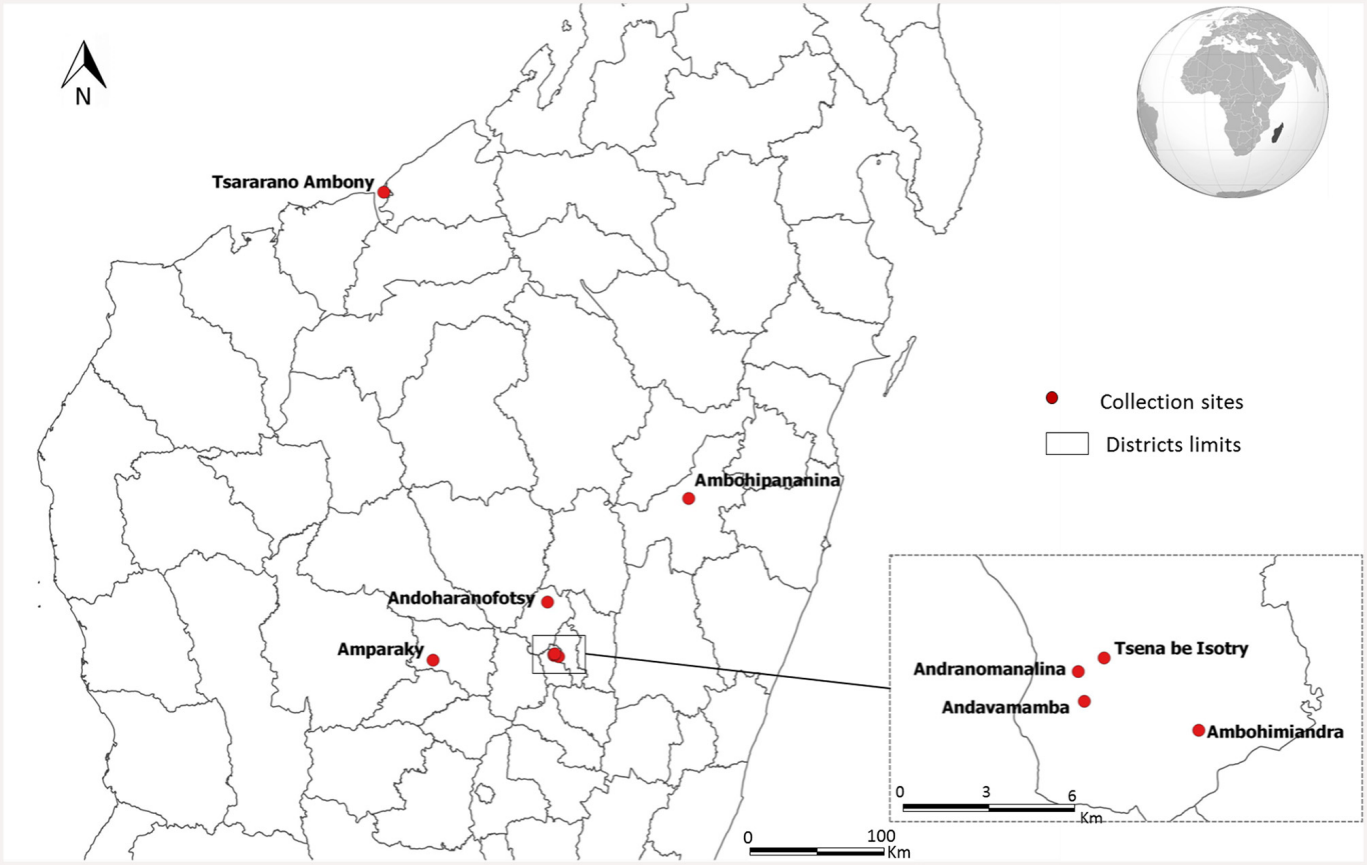
Map showing location of the eight sites where fleas were collected. Source: Institut Pasteur de Madagascar, OCHA.

A previous study reported the susceptibility of 32 populations of fleas to deltamethrin [27]. We chose to study eight populations from different geographical regions of Madagascar (Fig 1). Chosen populations were resistant fleas with mortality rates when exposed to deltamethrin of 2.5% to 65% [27]Flea populations (*X*. *cheopis*) were collected from the field and reared in an insectarium [27]. Briefly, rodents were trapped alive, fleas were combed into a large container, and fleas were reared in insectarium at 22–27°C and 75–80% relative humidity until having the sufficient number to perform bioassays. [27]. Fleas used in bioassays were subsequent generation of those collected in field.

### WHO bioassay

Bioassays were conducted on fleas populations according to the WHO protocol [28]. Ten adult fleas per tube were exposed to insecticide-impregnated paper (1.5 x 6 cm; Vector Control Research Unit, Penang, Malaysia) for specified times and at predetermined insecticide concentrations (Table 1). Each test was replicated at least four times for a total of 40 fleas per insecticide and per population. Negative controls were performed with paper only impregnated with the carrier of each insecticide family. LT50 (the time by which 50% of fleas were knocked down) were estimated for each insecticide during the diagnostic time. At the end of the exposure time and for all bioassays, the impregnated papers were removed and replaced by non-impregnated papers. Final mortality was recorded 24 hours after the beginning of exposure time. Susceptibility status was established according to the WHO guidelines for insecticide susceptibility test. Mortality rates of 98 to 100% indicated susceptibility, 80 to 98% tolerance or suspected resistance, and less than 80% resistance [29]. The test was not validated, and the data not included, if the negative control mortality rate was over 20%. The mortality rate was corrected with the Abbott formula [30] when control values were between 5% and 20%.

**Table 1:** Insecticides used in the bioassays with their concentration and the diagnostic exposure times.

**Insecticide**	**Family**	**Concentration (%)**	**Diagnostic time (hours)**
Alphacypermethrin	Pyrethroid	0.025	8
Lambdacyhalothrin	Pyrethroid	0.05	8
Etofenprox	Pyrethroid	0.5	8
Permethrin	Pyrethroid	0.75	8
Cyfluthrin	Pyrethroid	0.15	8
Deltamethrin	Pyrethroid	0.05	8
Bendiocarb	Carbamates	0.1	5
Propoxur	Carbamates	0.1	5
Malathion	Organophosohate	5	5
Fenitrothion	Organophosphate	1	5
DDT	Organochlorine	4	6
Dieldrin	Organochlorine	4	6

### Statistical analysis

Analysis of Variance (ANOVA) and Tukey’s b test were used to compare mortality rates. Mean LT50 and the standard errors for each flea population and for each insecticide were estimated with a binomial generalized linear model (glm) analysis. This glm including a probit function is a fitted model giving a prediction and a standard error at each response probability (p.model function with the package MASS). High mortality may not occur with some insecticides for some populations and therefore the LT50 would not be estimated (NE) Correlations between the mortality rates were calculated with Pearson tests (packages: corplot, Hmisc and ggplot2 to generate figures). Statistical analyses were done with R software (RStudio) [31].

## Results

### Mortality rate

**Fig 2:**
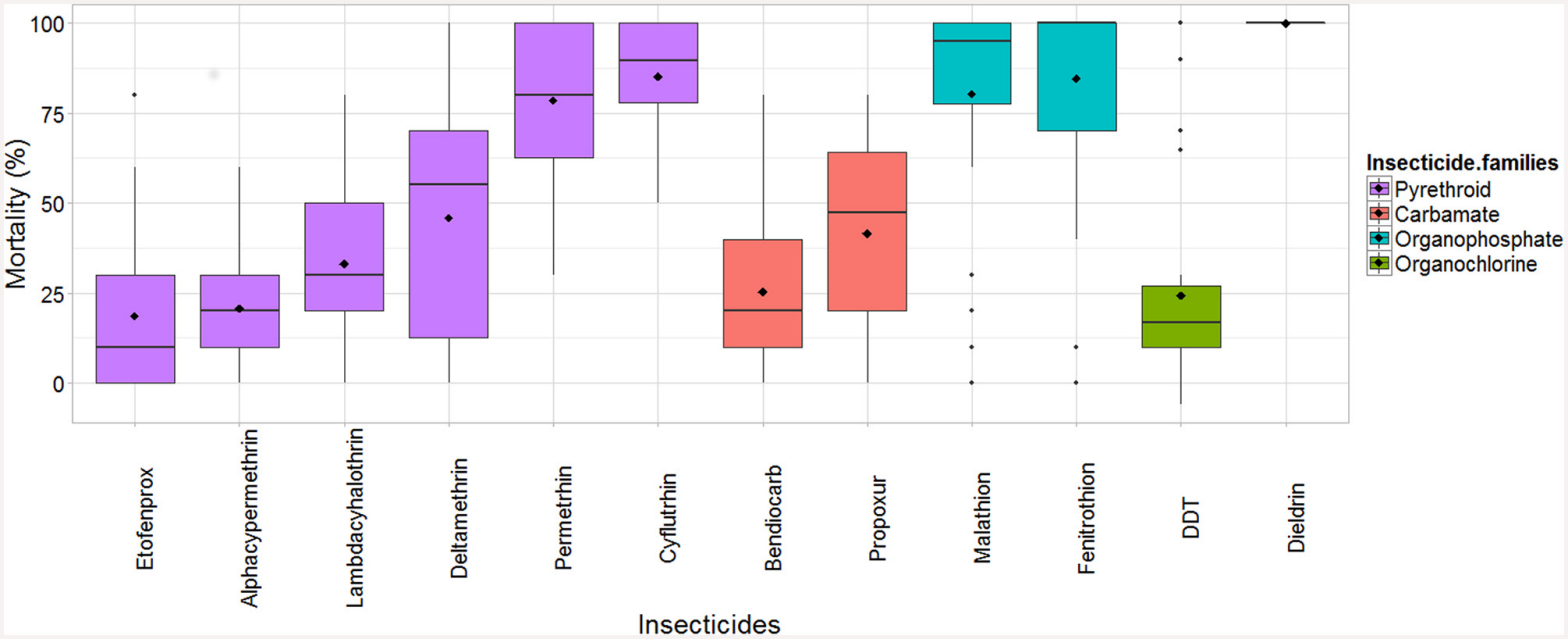
Box-and-whisker plot of mortality rate after 24 hours for each in secticide for all populations study sites. Black Diamond-shaped points inside the boxes are mean values. Horizontal bars in boxes are the 50^th^ percentiles (medians), and the bottom and the top of the box represent the 25^th^ and the 75^th^ percentiles, respectively. The two limits of vertical lines above and at the bottom of the box are the wiskers and represent the maximum and the minimum values of the data. Points outside the limit of vertical line are “outlier”, which are values outside 95% the confidence interval.

**Fig 3:**
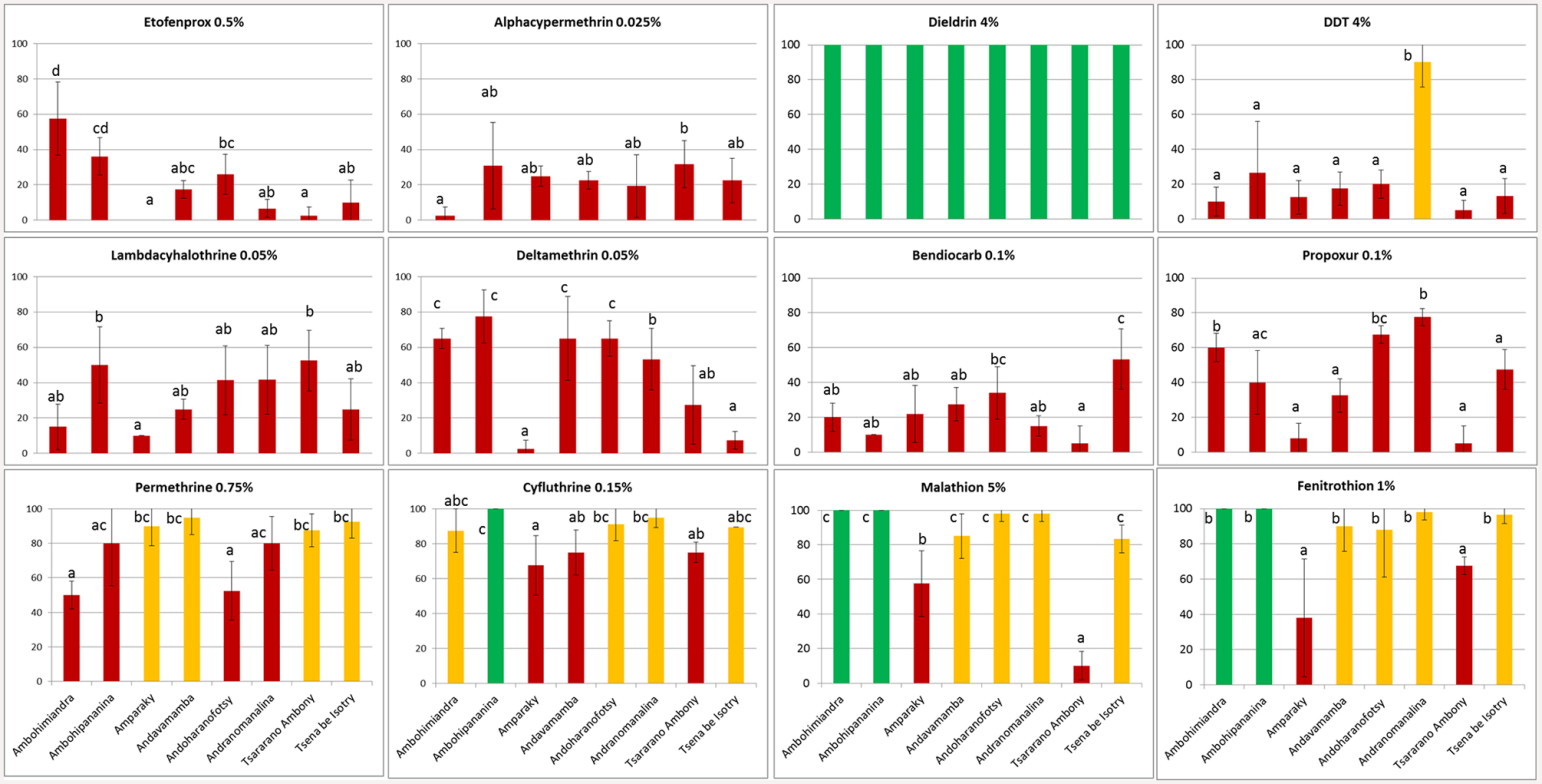
Mean of mortality rate (24 hours) per insecticide for each population. X-axis indicates populations and Y-axis indicates mortality rate in percent. Error bars represents standard errors. Diagrams color codes: in red are resistant populations, in yellow, tolerant and in green, susceptible according to WHO. Letters code (a, b, and c) above and on the side of each bar plot indicate significant difference between the mortality for each population according to the Tukey’s b test.

The mortality rate was different among insecticides and populations: mean mortality was significantly different according to populations (F value = 195.34, p < 0.0001) and insecticide (F value = 36.22, p < 0.0001). A strong correlation between insecticides and populations (F value = 9.10, p < 0.0001) was observed. Nonetheless, all populations were at least somewhat resistant to the six insecticides alphacypermethrin, lambdacyhalothrin, etofenprox, deltamethrin, bendiocarb and propoxur, with mortality rate ranging from 0 to 79% (Fig 2). Dieldrin was the only insecticide with 100% mortality rate for all flea populations (Fig 2). The resistance to DDT was substantial for most populations, with mortality rates varying between 5 and 26.4%, with the exception of one tolerant population, Andranomanalina, which had 90% mortality. Apart from the dieldrin, the highest mortality rates were observed for malathion, fenitrothion, cyfluthrin and permethrin (Fig 2). For these four insecticides, the susceptibility profiles were very different for each population (Fig 3). Almost the same resistance profile was observed for the organophoshates: two populations (Ambohimiandra and Ambohipananina) were susceptible with 100% mortality rate and fleas from Tsararano Ambony and Amparaky were both resistant to malathion and fenitrothion. Mortality induced by cyfluthrin ranged from 67.5 to 100% with one susceptible population (Ambohipananina) and four populations were tolerant. The mortality rate of the eight populations with permethrin varied between 50 and 95%, with two resistant populations.

### Lethal Time 50 and mortality rate

**Fig 4:**
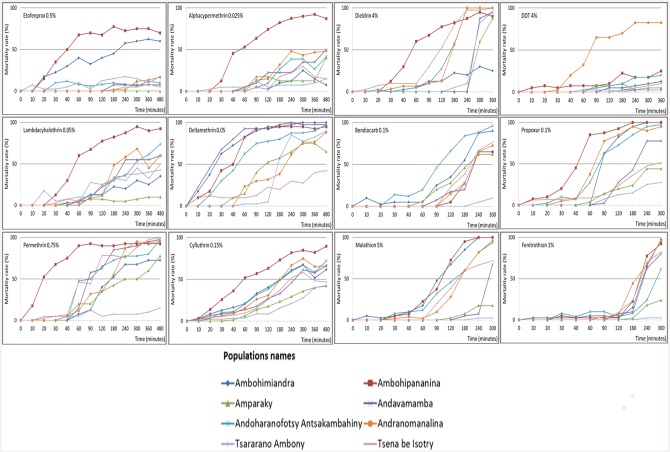
Mean mortality rates of flea populations per insecticides through the exposure time.

The curve profile, obtained during exposure time for each insecticide and for each station (Fig 4), and values of LT50 (S1 Table) were in concordance with the results obtained with the average mortality observed after 24 hours. Highly resistant population to insecticide had LT50 values longer than durations of exposure time. For Etofenprox, six tested populations had estimated LT50 > 500 minutes whereas exposure time was 480 minutes. These six populations had mortality below 30% after 24 hours. For DDT, no tested population reached LT50 until the exposure time (LT50 > 360 minutes), except for the tolerant population of Andranomanalina with a LT50 = 142 ± 8.80 minutes (Fig 4). Flea populations susceptible to cyfluthrin (Ambohipananina) had a LT50 equal to 21 ± 1.88 minutes and the tolerant ones had LT50 between 36 ± 4.58 and 128 ± 10.26 minutes. Even though the LT50 value of the population most resistant to cyfluthrin (Tsararano Ambony) was seven times higher than the value for the most susceptible population, the possible emergence of resistant individuals in tolerant populations could be suspected.

Similarly for malathion, susceptible population had an LT50 < 104 ± 5.14 minutes. LT50 was reached after 152 ± 6.81 to 285 ± 6.97 minutes for tolerant populations. Resistant populations’ LT50 values (Amparaky and Tsararano Ambony) exceeded the exposure time (300 minutes): LT50 were 370 ± 31.03 minutes and 464 ± 128.71 minutes, corresponding to 57.7% and 10% mean mortality after 24 hours, respectively.

Fig 4 illustrates the heterogeneity of response to insecticides amongst different flea populations. For example, in dieldrin trials, although 100% mortality rate was observed after 24 hours for every population, one population (Ambohimiandra) did not reach its LT50 value until the end of the exposure time (360 minutes). The LT50 value (425 ± 38.51 minutes), was 4 times higher than the minimal value obtained for dieldrin (99±6.84 minutes).

With propoxur, 100% of exposed fleas were knocked down before the exposure time was elapsed in two populations (Ambohimiandra and Ambohipnanina). These two populations had the shortest values of LT50 and lowest mortality rate values to propoxur after 24 hours (highly resistant populations).

### Mortality rate correlations between insecticides

**Fig 5:**
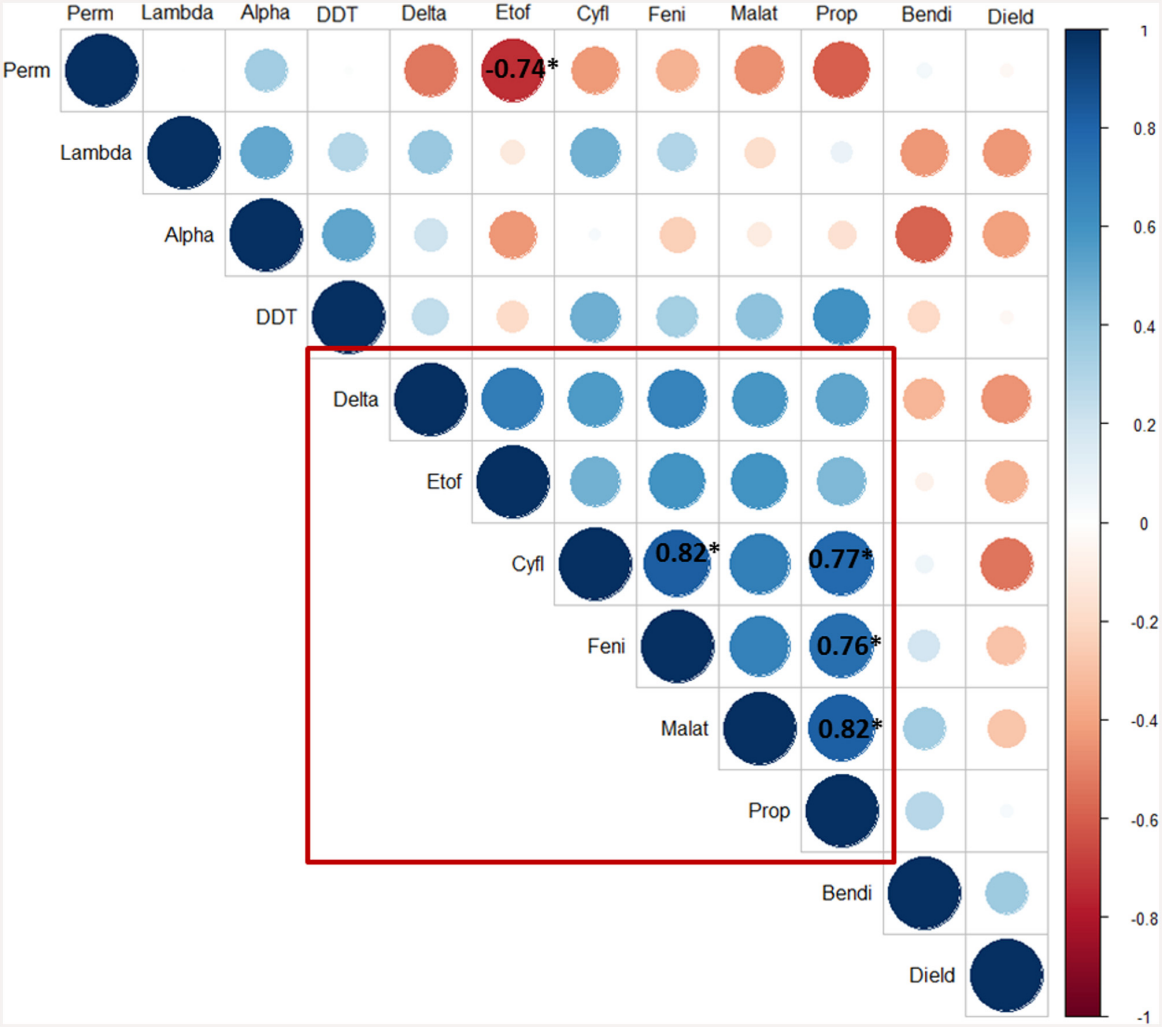
Schematic illustration of correlation between insecticide mortality rates (mean of 24h mortality per insecticide for all stations). Positive correlations are in blue and negative correlations are in red. The intensity of colors and the size of circles are proportional to the correlation coefficient. In the right, the color legend shows the correlation coefficient values with corresponding colors. Numbers followed by star are significant p values (p<0.05). Positive correlations between insecticides are represented surrounded with red rectangle.

Positive correlations were observed between deltamethrin, etofenprox, cyfluthrin, malathion, fenitrothion and propoxur (Fig 5), suggesting possible insecticide cross-resistance mechanisms in fleas. A strong negative correlation was observed between permethrin and etofenprox (r = -0.74, p<0.05). Significant correlations (p<0.05) were observed between fenitrothion and propoxur (r = 0.76, p = 0.03), propoxur and cyfluthrin (r = 0.77, p = 0.02), malathion and propoxur (r = 0.82, p = 0.01), and fenitrothion and cyfluthrin (r = 0.82, p = 0.01).

## Discussion

### *X*. *cheopis* resistance and insecticide uses in Madagascar

*X*. *cheopis* populations tested in this study were found highly resistant to DDT. Seven of eight populations showed final mortality rate less than 30% to DDT. These results reflected past observations of DDT resistance amongst *X*. *cheopis* populations from Madagascar, even though this product has not been used for many decades. The main argument raised to explain *X*.*cheopis* resistance to DDT worldwide was the extensive use of this insecticide in plague and malaria vector control [32–37]. In Madagascar, DDT was widely used against rat fleas since the 1940s [19]. DDT and pyrethroids were used in Indoor Residual Spraying (IRS) and long lasting insecticide impregnated nets against malaria vectors [38]. Furthermore, malaria vector treatment could have effect on flea vector resistance; in fact it was demonstrated elsewhere that insecticides used in IRS programs reduced flea loads on indoor rodents. [39]. However, in areas where malaria and plague are endemic, IRS treatment could have the potential to put selective pressure on fleas to develop resistance [40].

Dieldrin, an organochlorine insecticide, also saw widespread use in countries where plague occurred. Dieldrin was used in Madagascar during the period of DDT use, and *X*. *cheopis* was already described as resistant to dieldrin [21,26]. Insecticide susceptibility tests done in India showed that fleas resistant to DDT often were resistant to Dieldrin and other cyclodien insecticides [25, 29]. Yet, in our study, *X*. *cheopis* populations were all susceptible to this compound.

Pyrethrum was shown to have lethal effects on rat fleas before synthetic pyrethroids were used [41]. *X*. *cheopis* resistance to pyrethroid compounds (deltamethrin 0.025% and cyfluthrine 0.15%) was previously described in Madagascar [23,24]. Fleas from the Central Highlands of Madagascar were resistant to low concentrations of deltamenthrin (0.025%). Recently, 94% of studied populations were not sensitive to higher concentrations of deltamethrin (0.05%) [23,24,27]. The use of deltamethrin in plague control since the 1990s likely led to the development of resistant flea populations.

We present the first data illustrating resistance of *X*. *cheopis* populations to alphacypermethrin, lambdacyhalothrin and etofenprox, which were never used in mass vector control. This may suggest the involvement of cross-resistance mechanisms between these insecticides and those that were extensively used.

Organophoshates were also described as inducing resistance in rat flea populations. In India, *X*. *cheopis* was indicated as resistant to malathion and fenitrothion, although these compounds were never used in the study areas [36]. It was suggested that flea resistance to these compounds was associated with resistance to DDT [42]. Even if resistance to organophosphates was already described in some areas of Madagascar, the majority of populations studied presently showed less resistance to these compounds [21,43]. On the other hand, *X*. *cheopis* populations were previously described as susceptible to carbamate insecticides [24]; however, our study demonstrated a high resistance to propoxur and bendiocarb.

### Hypothesis on resistance mechanisms

Our results suggest resistance to all insecticides except dieldrin, which produced 100% mortality for all population. However, the LT50 values observed in one population (Ambohimiandra) suggest a progressive development of resistance to this compound. But dieldrin was banned in most of country worldwide because of its high toxicity in mammals and its bioaccumulation in the environment [44,45]. The use of dieldrin was suspended in Madagascar since 1993 [46]. However, other insecticide families having the same mode of action as dieldrin (antagonist of GABBA receptor) such as fiproles could be promising [10,47].

Six insecticides (alphacypermethrin, lambdacyhalothrin, etofenprox, deltamethrin, bendiocarb and propoxur) were relatively ineffective for flea control in all populations. Nonetheless, resistance level to the insecticides (permethrin, cyfluthrin malathion and fenitrothion) was very different among populations, suggesting different selection pressures. Hence, in this study, according to WHO thresholds, some insecticides were still efficient in some localities; thus, insecticides that induce resistance according WHO thresholds still may exhibit high performance in the field [12].

The different responses of populations to each insecticide reflect also the mode of action of insecticide molecules and the mechanism developed by insects to overcome toxic effects. Pyrethroids and DDT belong to a group of neurotoxic chemicals and share a similar mode of action distinct from other classes of insecticide. The studies on *kdr* mutation demonstrated the same mode of action of pyrethroids and DDT, which is the reduced target-site sensitivity of sodium channel [48]. Thus, the mechanism of resistance may not be specific to a particular insecticide family or group but the molecule structure of each insecticide can play important role.

For instance, the negative correlation between permethrin and etofenprox may involve the different effect induced by a Type I pyrethroid (permethrin) and a pseudo-pyrethroid (nonester pyrethroid) [49]. In addition, different levels of pyrethroid resistances were observed amongst populations. All studied populations were resistant to etofenprox alphacypermethrin, lambdacyhalothrin and deltamethrin; yet cyfluthrin and permethrin were effective in some localities. In a study of cross resistance amongst pyrethroids, cross resistance between 19 pyrethroid insecticides was assessed in bollworm moth, *Helicoverpa armigera* [50]. Cross resistance between pyrethroids seemed due to enhanced oxidative metabolism induced by pyrethroid with the same structure. The modification or replacement of any compound (aromatic compound) in the molecule structure could modify the susceptibility of the population [50].

Moreover, DDT and dieldrin belong to the oragnochlorine family, but their structures are very different, conferring different mode of action. The first attempt to elucidate cross resistance between chlorinated insecticides in *X*. *cheopis* was performed in 1974 [27]; a DDT-selected population was found to be resistant also to insecticides structurally related to DDT, and exhibited variable resistance to cyclodiene insecticides (such as dieldrin, endrin). But biochemical assays did not show significant difference between susceptible and resistant population. [32]. Other mechanisms like *Rdl* mutation can confer resistance to cyclodiene like dieldrin [47].

Furthermore, the correlations between deltamethrin, etofenprox, propoxur, and between cyfluthrin, malathion and fenitrothion, may be explained by the same mechanism of resistance developed by *X*. *cheopis* for these insecticides. The absence of references on this topic in Siphonaptera illustrates a need for further research on insecticide resistance mechanisms in fleas.

### Perspectives on improving fight against fleas vector of plague

Efforts can be undertaken before each epidemic season in order to control the proliferation of vectors and host, such as live rat mass trapping, promotion of rat proofing in houses and environment sanitation [15]. One available method could be the use of insecticide bait box, combining insecticide and delayed toxicity rodenticide [51–55]. The objective is to kill fleas on rodents before the action of the rodenticide. Instead of insecticide dusting in household, the quantity of insecticide is reduced because the insecticide bait box is more focused on fleas harbored by rodent with the host acting as a vehicle for the insecticide, carrying it to its nest. In the same line of thought, the feasibility of “insecticide delivery tubes” in reducing flea loads was studied on commensal rodents, capitalizing on the knowledge of their behavior [56]. Similarly, using rodent bait containing systemic insecticide could be a new avenue for combating or at least, reducing fleas load on rodents in plague endemic area during inter-epidemic season [57]. Besides, novel approaches to fighting vector limiting the use of chemical insecticides should be explored in order to avoid insecticide resistance [58,59]. For instance, research must be undertaken in the way to better understand the interaction between the vector, the pathogen and the insect microbiome. The strategy is based on introduction of microorganism which may affect the insect lifespan or the ability to transmit the pathogen [59–61]. Furthermore, research on bio pesticide is already ongoing with the use of entomopathogen fungi to reduce the survival of flea larvae [62].

### Recommendations for public health concern

The main finding in this study is that *X*. *cheopis* populations developed resistance mechanisms to the insecticide families most used in vector control. The description of phenotypic resistance to insecticides is important to help practitioners choose the most efficient strategy in pest management. Hence, in a public health context, insecticide susceptibility status of fleas in each plague risk area may be monitored periodically to conduct more focused and adapted flea control. However information available on the mechanism of resistance and cross-resistance about *X*. *cheopis* is scarce or nonexistent. Research must be done to understand the mechanisms conferring resistance to insecticides in plague vectors such *X*. *cheopis*.

## Supporting Information

S1 Table Table of the values of LT50 in minutes and standard error values for each insecticide and station. (XLSX)

## References

[1] World Health Organization. Vector-borne diseases. Fact sheet N°387. 2014; Available: www.who.int/mediacentre/factsheets/fs387/en/

[2] Dantas-Torres F, Otranto D. Dogs, cats, parasites, and humans in Brazil: opening the black box. Parasit Vectors. 2014;7: 22 Available: http://www.parasitesandvectors.com/content/7/1/22 10.1186/1756-3305-7-22 24423244 PMC3914713

[3] Nauen R. Insecticide resistance in disease vectors of public health importance Pest Manag Sci. John Wiley & Sons, Ltd.; 2007;633: 628–633. 10.1002/ps.1406 17533649

[4] Brogdon W, McAllister J. Insecticide resistance and vector control. Emerg Infect Dis. 1998; Available: http://www.ncbi.nlm.nih.gov/pmc/articles/PMC2640263/10.3201/eid0404.980410 PMC2640263 9866736

[5] Rivero A, Vézilier J, Weill M, Read AF, Gandon S. Insecticide Control of Vector-Borne Diseases: When Is Insecticide Resistance a Problem? PLoS Pathog. 2010;6: e1001000 10.1371/journal.ppat.1001000 20700451 PMC2916878

[6] Corbel V, Hougard J-M, N’Guessan R, Chandre F. Evidence for selection of insecticide resistance due to insensitive acetylcholinesterase by carbamate-treated nets in Anopheles gambiae s.s. (Diptera: Culicidae) from Côte d’Ivoire. J Med Entomol. 2003;40: 985–988. 14765680 10.1603/0022-2585-40.6.985

[7] Lotfy W. Current perspectives on the spread of plague in Africa Res Rep Trop Med. Dove Press; 2015;Volume 6: 21.

[8] Neerinckx S, Bertherat E, Leirs H. Human plague occurrences in Africa: an overview from 1877 to 2008. Trans R Soc Trop Med Hyg. 2010;104: 97–103. Available: http://trstmh.oxfordjournals.org/content/104/2/97.short 10.1016/j.trstmh.2009.07.028 19716148

[9] Hemingway J, Ranson H. Insecticide Resistance in Insect Vectors of Human Disease. Annu Rev Entomol. 2000;45: 371–391. 10761582 10.1146/annurev.ento.45.1.371

[10] Sparks T, Nauen R. IRAC: Mode of action classification and insecticide resistance management. Pestic Biochem Physiol. 2014; Available: http://www.sciencedirect.com/science/article/pii/S0048357514002272 10.1016/j.pestbp.2014.11.014 26047120

[11] Sridhar V, Lokeshwari D. Insecticide resistance management: reflections and way forward. Curr Sci. 2014;107 Available: https://scholar.google.fr/scholar?hl=fr&q=Insecticide+resistance+management%3A+reflections+and+way+forward&btnG=&lr=#0

[12] Gnanguenon V, Agossa FR, Badirou K, Govoetchan R, Anagonou R, Oke-Agbo F, et al Malaria vectors resistance to insecticides in Benin: current trends and mechanisms involved. Parasit Vectors. 2015;8: 1–14. 10.1186/s13071-015-0833-2 25886599 PMC4395909

[13] Breu F, Guggenbichler S, Wollmann J. World Health Statistics 2013. [Internet]. Vasa. 2013.

[14] Andrianaivoarimanana V, Kreppel K, Elissa N, Duplantier J-MM, Carniel E, Rajerison M, et al Understanding the persistence of plague foci in Madagascar. PLoS Negl Trop Dis. 2013;7: e2382 10.1371/journal.pntd.0002382 24244760 PMC3820717

[15] World Health Organization. Operational guidelines on plague surveillance, diagnosis, prevention and control [Internet]. New Delhi: Regional Office for South-East Asia; 2009 Available: https://scholar.google.fr/scholar?q=Operational+Guidelines+on+Plague+Surveillance%2C+Diagnosis%2C+Prevention+and+Control&btnG=&hl=fr&as_sdt=0%2C5#0

[16] World Health Organization. Pesticides and their application for the control of vectors and pets of public health importance [Internet]. Sixth edit. Curtis C.F. (London School of Hygiène and Tropical Medecine), editor. Geneva, Switzerland: Department of Control of Neglected Tropical Diseases WHO Pesticide evaluation scheme (WHOPES); 2006 Available: http://apps.who.int/iris/handle/10665/69223

[17] Chanteau S, Ratsitorahina M, Rahalison L, Rasoamanana B, Chan F, Boisier P, et al Current epidemiology of human plague in Madagascar. Microbes Infect. 2000;2: 25–31. 10.1016/S1286-4579(00)00289-6 10717537

[18] World Health Organization. Interregional meeting on prevention and control of plague [Internet] Global Alert and Response (GAR). Antananarivo, Madagascar: World Health Organisation; 2006 Available: http://www.who.int/csr/resources/publications/WHO_HSE_EPR_2008_3w.pdf

[19] Brygoo ER. Epidémiologie de la peste à Madagascar. Arch Inst Pasteur Madagascar. 1966;35 Available: http://www.pasteur.mg/IMG/pdf/Arch_lnst_Pasteur_Madagascar_1966_35_1_4-147.pdf

[20] Coulanges P, Randrianantoanina E. Résistance exceptionnelle aux insecticides de puces pestigènes malgaches. Bull la Soc Pathol Exot. 1984;77: 705–711. 6525729

[21] Coulanges P, Randrianantoanina E. Résistance des puces pestigènes malgaches aux insecticides organochlores, organophosphores et aux carbamates. Arch Inst Pasteur Madagascar. 1984;51: 253–260. 6534290

[22] Fontenille D, Coulanges P. Notes sur la sensibilité des puces Xenopsylla cheopis de la région d’Antananarivo à la Déltamethrine et au Propoxur. Arch Inst Pasteur Madagascar. 1987;53: 249–259. 3451707

[23] Ratovonjato J. Sensibilité de Xenopsylla cheopis aux insecticides en milieu urbain à Madagascar. Arch Inst Pasteur Madagascar. 1998;64: 25–28.

[24] Ratovonjato J, Duchemin J-B, Duplantier J-M, Chanteau S. Xenopsylla cheopis (Siphonaptera: Xenopsyllinae), puces des foyers ruraux de peste des Hautes Terres malgaches: niveau de sensibilité au DDT, aux pyréthrinoïdes et aux carbamates après 50 années de lutte chimique. Arch Inst Pasteur Madagascar. 2000;66: 9–12. 12463026

[25] Ratovonjato J, Duchemin J. Evaluation de l’effet du Knox-Out^®^ microencapsulé VO 240 et de la K-othrine^®^ poudre sur les puces des rats de deux villages de la région de Betafo. Arch Inst Pasteur Madagascar. 2001; Available: http://www.pasteur.mg/IMG/pdf/knoxout.pdf 12471748

[26] Coulanges P. Etude de X. Cheopis et S. Fonquerniei, puces pestigènes malgaches: mise en évidence de leur résistance au DDT, Dieldrin et Malathion. Arch Inst Pasteur Madagascar. 1982;49: 171–191. Available: http://cat.inist.fr/?aModele=afficheN&cpsidt=9310285 7186791

[27] Boyer S, Miarinjara A, Elissa N. Xenopsylla cheopis (Siphonaptera: Pulicidae) Susceptibility to Deltamethrin in Madagascar. PLoS One. 2014;9: e111998 Available: <Go to ISI>://WOS:000344402000135 10.1371/journal.pone.0111998 25369291 PMC4219825

[28] World Health Organization. Report of WHO Expert Committee: Resistance of Vectors and Reservoirs of disease to pesticide:twenty-second report of the WHO Expert Committee on Insecticides [meeting held in Geneva from 16 to 23 September 1975] [Internet]. World Health Organization technical report series; no. 585. Geneva: WHO; 1976. Available: http://apps.who.int/iris/handle/10665/41190#sthash.tuGP3Hvr.dpuf 817518

[29] Word Health Organization. The technical basis for coordinated action against insecticide resistance. 2011.

[30] Abbott W. A method of computing the effectiveness of an insecticide. J econ Entomol. 1925; Available: http://demoportal.mans.edu.eg/scifac/images/files/abstracts/chemistry/MamdouhAbdelMogib.pdf 3333059

[31] R Core Team. R: A Language and Environment for Statistical Computing. Vienna, Austria: R Foundation for Statistical Computing; 2014.

[32] Kalra R, Joshi G. Studies on the Insecticide Resistance in Rat Fleas, Xenopsylla cheopis (Roth.). Botyu-Kagaku. 1974;39: 110–115. Available: http://repository.kulib.kyoto-u.ac.jp/dspace/handle/2433/158862

[33] Kilpatrick JW, Fay RW. DDT-Resistance Studies with the Oriental Rat Flea. J Econ Entomol. The Oxford University Press; 1952;45: 284–288.

[34] Mourya D. Present Insecticide Susceptibility Status Of Xenopsylla cheopis From Beed District, Maharashtra State, India. Entomon. 1998;23: 211–217.

[35] Patel TB, Bhatia SC, Deobhankar RB. A confirmed case of DDT-resistance in Xenopsylla cheopis in India. Bull World Health Organ. 1960;23: 301–312. Available: http://www.pubmedcentral.nih.gov/articlerender.fcgi?artid=2555587&tool=pmcentrez&rendertype=abstract 14430833 PMC2555587

[36] Renapurkar D. Distribution and insecticide resistance of the plague flea Xenopsylla cheopis in Maharashtra State, India. Med Vet Entomol. 1990; Available: http://onlinelibrary.wiley.com/doi/10.1111/j.1365-2915.1990.tb00264.x/abstract 10.1111/j.1365-2915.1990.tb00264.x 2132973

[37] Shalaby AM. Susceptibility Status of the rat flea Xenopsylla cheopis Roths. (Pulicidae) to DDT, Gamma BHC and Dieldrin, in Lybia. Zeitschrift für Angew Entomol. 1971;69: 64–71.

[38] Ratovonjato J, Randrianarivelojosia M, Rakotondrainibe ME, Raharimanga V, Andrianaivolambo L, Le Goff G, et al Entomological and parasitological impacts of indoor residual spraying with DDT, alphacypermethrin and deltamethrin in the western foothill area of Madagascar. Malar J. 2014;13: 18 Available: http://www.biomedcentral.com/content/pdf/1475-2875-13-21.pdf 24423246 10.1186/1475-2875-13-21 PMC3906765

[39] Borchert JN, Eisen RJ, Atiku LA, Delorey MJ, Mpanga JT, Babi N, et al Efficacy of Indoor Residual Spraying Using Lambda-Cyhalothrin for Controlling Nontarget Vector Fleas (Siphonaptera) on Commensal Rats in a Plague Endemic Region of Northwestern Uganda. J Med Entomol. 2012;49: 1027–1034. 23025183 10.1603/me11230

[40] Ames A. DDT and pyrethroid resistance in Xenopsylla cheopis (Rothschild), the oriental rat flea in northern Uganda [Internet]. Colorado State University. 2011. Available: http://digitool.library.colostate.edu/exlibris/dtl/d3_1/apache_media/L2V4bGlicmlzL2R0bC9kM18xL2FwYWNoZV9tZWRpYS8xMjM2MjM=.pdf

[41] Gratz N, Traub R, Starcke H. Problems and developments in the control of flea vectors of disease. Proceedings of the International Conference on Fleas, Ashton Wold, Peterborough, UK, 21–25 June 1977. AA Balkema. Peterborough, UK; 1980.

[42] Shyamal B, Ravi Kumar R, Sohan L, Balakrishnan N, Veena M, Shiv L. Present susceptibility status of rat flea Xenopsylla cheopis (Siphonaptera: Pulicidae), vector of plague against organochlorine, organophosphate and synthetic pyrethroids 1. The Nilgiris District, Tamil Nadu, India. J Commun Dis. 2008;40: 41–5. Available: http://europepmc.org/abstract/med/19127668 19127668

[43] Coulanges P, Clerc Y, Randrianantoanina E. X. cheopis and S. fonquerniei, plague-carrying Malagasian fleas—demonstration of their resistance to DDT, dieldrin and malathion. Arch Inst Pasteur Madagascar. 1982;49: 171 7186791

[44] Ackerman LB. Overview of human exposure to dieldrin residues in the environment and current trends of residue levels in tissue. Pestic Monit J. 1980;14: 64–9. Available: http://europepmc.org/abstract/med/7232105 7232105

[45] Kanthasamy AG, Kitazawa M, Kanthasamy A, Anantharam V. Dieldrin-induced neurotoxicity: relevance to Parkinson’s disease pathogenesis. Neurotoxicology. 2005;26: 701–19. 16112328 10.1016/j.neuro.2004.07.010

[46] Ministère d’Etat à l'Agriculture et au Développement Rural. ARRETE N° 6225/93 portant suspension et restriction d’utilisation de quelques produits agropharmaceutiques [Internet]. Available: http://faolex.fao.org/docs/pdf/mad148368.pdf

[47] Kristensen M. Cross-resistance between dieldrin and fipronil in German cockroach (Dictyoptera: Blattellidae). J Econ Entomol. 2005; Available: http://jee.oxfordjournals.org/content/98/4/1305.abstract 10.1603/0022-0493-98.4.1305 16156584

[48] Liu N. Insecticide Resistance in Mosquitoes: Impact, Mechanisms, and Research Directions. Annu Rev Entomol. 2015; Available: http://www.annualreviews.org/doi/abs/10.1146/annurev-ento-010814-020828 10.1146/annurev-ento-010814-020828 25564745

[49] Schleier JJ III, Peterson RKD. Pyrethrins and Pyrethroid Insecticides In: Lopez Oscar, Fernfmdez-Bolafios Jose G., editors. Green Trends in Insect Control. Royal Society of Chemistry www.rsc.org; 2011 pp. 94–131. 10.1039/9781849732901-00094

[50] Tan J, McCaffery A. Efficacy of various pyrethroid structures against a highly metabolically resistant isogenic strain of Helicoverpa armigera (Lepidoptera: Noctuidae) from China. Pest Manag Sci. 2007; Available: http://onlinelibrary.wiley.com/doi/10.1002/ps.1419/full 10.1002/ps.1419 17685437

[51] Kartman L, Lonergan R. Wild-rodent-flea control in rural areas of an enzootic plague region in Hawaii: A preliminary investigation of methods. Bull World Health Organ. 1955;13: 49 Available: http://www.ncbi.nlm.nih.gov/pmc/articles/PMC2538036/ 13260882 PMC2538036

[52] Barnes A, Kartman L. Control of plague vectors on diurnal rodents in the Sierra Nevada of California by use of insecticide bait-boxes. J Hyg (Lond). 1960; Available: http://journals.cambridge.org/abstract_S0022172400038468 10.1017/s0022172400038468 PMC2134376 13687091

[53] Ratovonjato J, Duchemin JB, Duplantier JM, Rahelinirina S, Soares JL, Rahalison L, et al Lutte contre la peste à Madagascar: évaluation de l’efficacité des boîtes de Kartman en milieu urbain. Arch Inst Pasteur Madagascar. Institut Pasteur de Madagascar; 2003;69: 41–45. Available: http://cat.inist.fr/?aModele=afficheN&cpsidt=16186366 15678815

[54] Kartman L. An insecticide-bait-box method for the control of sylvatic plague vectors. J Hyg (Lond). 1958; Available: http://journals.cambridge.org/abstract_S0022172400037967 10.1017/s0022172400037967 PMC2218086 13611242

[55] Kartman L. Further observations on an insecticide-bait-box method for the control of sylvatic plague vectors; effect of prolonged field exposure to DDT powder. J Hyg (Lond). 1960; Available: http://journals.cambridge.org/abstract_S0022172400038171

[56] Boegler KA, Atiku LA, Mpanga JT, Clark RJ, Delorey MJ, Gage KL, et al Use of Insecticide Delivery Tubes for Controlling Rodent-Associated Fleas in a Plague Endemic Region of West Nile, Uganda. J Med Entomol. 2014;51: 1254–1263. Available: <Go to ISI>://WOS:000345123800022 10.1603/ME14083 26309315 PMC4599340

[57] Borchert JN, Enscore RE, Eisen RJ, Atiku LA, Owor N, Acayo S, et al Evaluation of rodent bait containing imidacloprid for the control of fleas on commensal rodents in a plague-endemic region of northwest Uganda. J Med Entomol. 2010;47: 842–850. 20939379 10.1603/me09221

[58] Raharimalala FN, Boukraa S, Bawin T, Boyer S, Francis F. Molecular detection of six (endo-) symbiotic bacteria in Belgian mosquitoes: first step towards the selection of appropriate paratransgenesis candidates. Parasitol Res. 2015; 10.1007/s00436-015-4873-5 26670313

[59] Beard C. Bacterial symbiosis in arthropods and the control of disease transmission. Emerg Infect Dis. 1998;4: 581 Available: http://www.ncbi.nlm.nih.gov/pmc/articles/PMC2640264/ 9866734 10.3201/eid0404.980408 PMC2640264

[60] Leitner W, Wali T. Arthropod Vectors and Disease Transmission: Translational Aspects. PLoS Negl Trop Dis. 2015;9 Available: http://www.ncbi.nlm.nih.gov/pmc/articles/PMC4652900/10.1371/journal.pntd.0004107 PMC4652900 26583380

[61] Erickson D. Bacterial communities associated with flea vectors of plague. J Med Entomol. 2009;46: 1532–1536. Available: http://jme.oxfordjournals.org/content/46/6/1532.abstract 19960708 10.1603/033.046.0642

[62] Mnyone L, Ng’habi K. Entomopathogenic fungi, Metarhizium anisopliae and Beauveria bassiana reduce the survival of Xenopsylla brasiliensis larvae (Siphonaptera: Pulicidae). Parasit Vectors. 2012;5: 204 Available: http://www.biomedcentral.com/content/pdf/1756-3305-5-204.pdf 10.1186/1756-3305-5-204 22992264 PMC3468376

